# Plant Robotics for Sustainable and Environmentally Friendly Robots: Insights from Actuation Characteristics

**DOI:** 10.1002/advs.202512896

**Published:** 2025-11-14

**Authors:** Kazuya Murakami, Misao Sato, Yu Ikeda, Tatsuhiro Horii, Yukari Nagatoshi, Miki Fujita, Toshinori Fujie, Yasunari Fujita, Jun Shintake

**Affiliations:** ^1^ Department of Mechanical and Intelligent Systems Engineering The University of Electro‐communications 1‐5‐1 Chofugaoka Chofu Tokyo 182‐8585 Japan; ^2^ School of Life Science and Technology Institute of Science Tokyo B‐50 4259 Nagatsuta‐cho Midori‐ku Yokohama 226‐8501 Japan; ^3^ Research Center for Autonomous Systems Materialogy (ASMat) Institute of Integrated Research (IIR) Institute of Science Tokyo B‐50 4259 Nagatsuta‐cho Midori‐ku Yokohama 226‐8501 Japan; ^4^ Biological Resources and Post‐harvest Division Japan International Research Center for Agricultural Sciences (JIRCAS) 1‐1 Owashi Tsukuba Ibaraki 305‐8686 Japan; ^5^ Food Program Japan International Research Center for Agricultural Sciences (JIRCAS) 1‐1 Owashi Tsukuba Ibaraki 305‐8686 Japan; ^6^ Graduate School of Life Environmental Science University of Tsukuba 1‐1‐1 Tennodai Tsukuba Ibaraki 305‐8577 Japan; ^7^ Mass Spectrometry and Microscopy Unit RIKEN Center for Sustainable Resource Science Technology Platform Division 3‐1‐1 Koyadai Tsukuba Ibaraki 305‐0074 Japan

**Keywords:** actuators, biohybrid, plant, plant robotics, sustainability

## Abstract

Robots play an ever‐expanding role in society by performing a broad range of tasks. However, there are growing concerns about their environmental sustainability, as many conventional robotic systems rely on materials that are neither renewable nor degradable. Consequently, significant efforts are being made to develop eco‐friendly robots built from sustainable and biodegradable materials. In this context, plants represent a promising direction, as the biomaterials composing plants are biodegradable, and their inherent multifunctionality as living organisms, including sensing, actuation, energy harvesting, and self‐healing, makes them strong candidates for realizing biodegradable robotic systems. Moreover, they are abundant and renewable resources. Recent studies have demonstrated plant‐based robotic systems that harness some of these features, helping to establish plant robotics as an emerging research field. Among the many functions plants offer, actuation is pivotal, as it enables physical robotic motion, such as locomotion and grasping, which substantially broadens the potential applications of plant robots. Focusing on plant movement, this article reviews key plant species and their behaviors through the perspective of actuation characteristics. It also examines the current landscape of plant‐based robotic systems and outlines future research directions in this rapidly growing field.

## Graphical abstract

Plants are promising materials for building sustainable and eco‐friendly robots due to their inherent multifunctionality, with actuation playing a crucial role. This article focuses on the physical movements of plants and, from the perspective of actuation characteristics, explores representative plant species and their behaviors, the current state of plant robots, and future prospects for this emerging field.

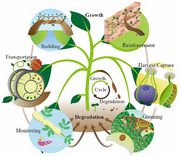

## 1 Introduction

Robots that decompose and naturally integrate into the environment at the end of their task or operational life represent an ideal form of environmentally harmonized intelligent machines due to their inherent sustainability and eco‐friendliness. One promising pathway toward realizing such systems is the use of biodegradable materials as the main building materials for the robots, which can break down and return to the soil. In line with this approach, the field of soft robotics,^[^ ^1^, ^2^, ^3^, ^4^, ^5^, ^6^, ^7^ ^]^ in which robotic bodies are often formed from continuous structures composed of a single material, has seen growing interest in the development of robots, actuators, sensors, and other associated elements made from biodegradable materials.^[^ ^8^, ^9^, ^10^ ^]^

Plants are especially promising among the various candidates for sustainable and environmentally friendly materials for robotic systems. This is not only because they are naturally biodegradable and follow cyclical life cycles from germination to death and regeneration in natural environments, but also because they inherently possess functions that are essential for intelligent machines and robots. For example, a sunflower tracks the sun on a clear day and rotates its stem, plants undergo physical shape changes in response to environmental stimuli. This behavior naturally integrates both sensing and actuation, powered by photosynthesis as the energy source. If such stimuli‐responsive behavior were interpreted as a form of information processing, plants could be viewed as functional systems analogous to controllers or computational units. It is also well known that plants possess self‐healing capabilities. While these functionalities have motivated the field of plant‐inspired robotics, which aims to replicate plant functions using engineered materials,^[^ ^11^, ^12^, ^13^ ^]^ this perspective article distinguishes plant robotics as a distinct approach that directly integrates living plants into electromechanical systems, harnessing their innate capabilities, to create so‐called biohybrid systems. The functional traits of plants and their potential for both plant robotics and plant‐robot biohybrids are well summarized in review papers in the literature.^[^ ^14^, ^15^ ^]^ These ongoing research efforts are expected to lead the development of fully biodegradable plant‐based robotic systems and, ultimately, to the creation of “true” plant robots made entirely from living plants.

**Figure 1:**
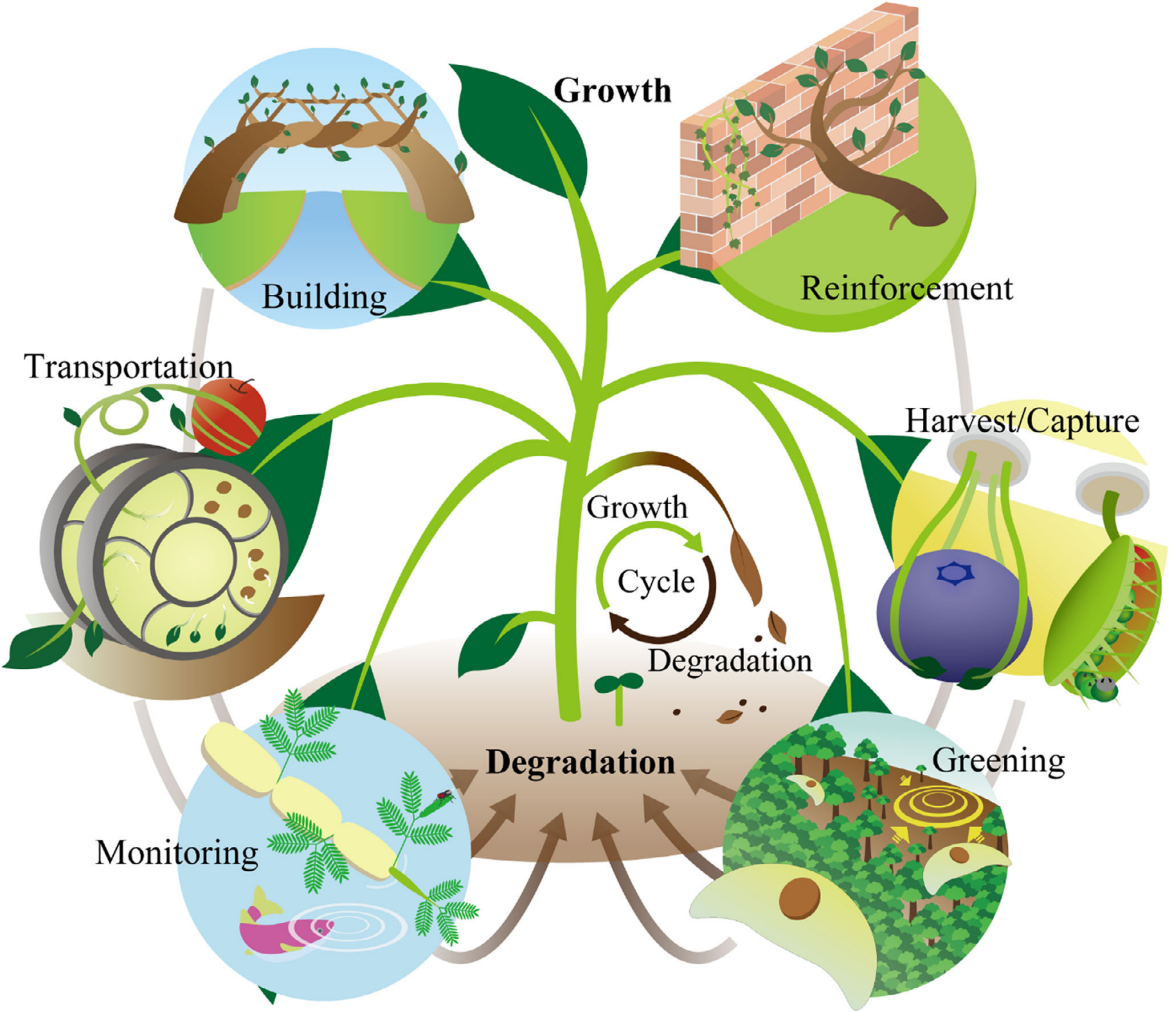
Potential applications of plant robots utilizing physical movements. Through natural processes of growth and degradation, combined with functionalities analogous to robotic elements such as actuation and sensing, plants can fulfill diverse roles – including the structural formation, reinforcement of artificial buildings, object harvesting and capture, environmental greening, monitoring, and transportation. This multifunctionality, integrated into a self‐sustaining life cycle, highlights the potential of plants as dynamic platforms for developing sustainable and eco‐friendly robotic systems.

To realize such plant robots, actuators that generate physical motion are an essential component forming the foundation of robotic systems. Through actuation, robots are able to interact with their environment and perform tasks. This perspective also offers new insight into plants, which are commonly perceived as static due to their rooted nature. However, as demonstrated in several studies later in this paper, plants can be engineered to gain mobility and exhibit dynamic behavior. Plants naturally change shape as they grow and can move in response to external stimuli like light or electrical signals. If these movements can be utilized as actuation mechanisms, then, combined with the natural diversity of plants, it becomes possible to envision robots capable of executing a wide range of motions and performing diverse tasks, as illustrated in **Figure** **1**. This article focuses on the physical movements of plants and discusses, from the standpoint of actuation characteristics, representative plants and their behaviors, the current state of plant‐based robotic systems, and the outlook for this emerging field: plant robotics.

## 2 Moving Plants

It is important to note that most plants exhibit some sort of movement, which can broadly be categorized. These include irreversible growth movements, reversible turgor movements, which are reversible and driven by changes in cell turgor pressure, and passive movements, such as the response of the hygroscopic cell walls to changes in humidity. Among these, movement of leaves, flowers, branches, or roots in response to the direction of a stimulus is called tropism. This type of movement is typically driven by growth. Conversely, movements that occur rapidly and independently of stimulus direction are called nastic movements. Most nastic movements are driven by turgor changes, although some are triggered by growth. In addition to these types of movements, plants use various strategies to disperse seeds over long distances, helping expand their reproductive range. This seed dispersal is also considered a type of plant movement.

To apply plant movements to robots or devices, it is essential to understand their input‐output relationships. This section systematically classifies key plant movements and introduces their respective input‐output relationships and actuation characteristics. Most plants exhibit a wide range of movements; those that have been quantitatively analyzed to date are presented in **Table** **1**. The table summarizes, for each listed species, the primary stimulus, driving force, movement type, deformation behavior, and measured mechanical outputs, such as displacement, force, and speed. The following subsections will provide a detailed explanation of the plant species listed in Table 1.

**Table 1:** Actuation characteristics and related features of representative plants.

Name of plant	Moving part	Driving Force	Movement Type	Mechanism	Stimulus (Input)	Deformation type (Output)	Displacement	Actuation time	Speed	Force	Growing time before actuation is possible	Size	Life cycle
Radish sprout (*Raphanus sativus var.Longipinnatus*)	Stem	growth	tropism	growth, gravitropism, phototropism	gravity, light, etc.	elongation	16.3‐46.4 mm (40 h)^[^ ^20^ ^]^	N/A	0.4‐1.2 mm/h^[^ ^20^ ^]^	31.8‐35.4 mN (40mm), 60.5‐97.5 mN (seed)^[^ ^20^ ^]^	2 days (80% germination rate)^[^ ^27^ ^]^	over 7.6 cm^[^ ^20^ ^]^	annual
Dandelion (*Taraxacum officinale*)	stem	growth	tropism	growth, gravitropism, phototropism	gravity, light, etc.	elongation	5‐50 cm^[^ ^16^ ^]^	N/A	N/A	2‐3 N^[^ ^30^ ^]^	8‐16 days (80% germination rate)^[^ ^28^ ^]^	5‐50 cm in height^[^ ^16^ ^]^	perennial^[^ ^16^ ^]^
Moso bamboo (*Phyllostachys edulis*)	culm internodes	growth	tropism	growth, gravitropism, phototropism	gravity, light, mechanical load	elongation	9.67‐17.5 m^[^ ^21^ ^]^	35‐40 days^[^ ^33^ ^]^	114.5 cm/day (max),^[^ ^31^ ^]^ 30–100 cm/day (during peak growth)^[^ ^32^ ^]^ stages)	N/A	N/A	9.67‐17.5 m in height, 5.10‐17.2 cm in diameter^[^ ^21^ ^]^	perennial
Common bean (*Phaseolus vulgaris*)	leaflet	turgor	nastic movement	photonasty, nyctinasty	light, circadian clock	bending	89°^[^ ^34^ ^]^	7 h^[^ ^34^ ^]^	13° /h^*1^	N/A	N/A	over 2 m in height^[^ ^17^ ^]^	annual^[^ ^17^ ^]^
Black locust (*Robinia pseudoacacia*)	leaflet	turgor	nastic movement	photonasty, nyctinasty	light, circadian clock	bending	102°^[^ ^35^ ^]^	9 h^[^ ^35^ ^]^	11°/h^*1^	N/A	N/A	up to 20 m in height, leaves 20–35 cm^[^ ^22^ ^]^	perennial
Sensitive plant (*Mimosa pudica*)	petiole	turgor	nastic movement	photonasty, nyctinasty	light, circadian clock	bending (rise)	N/A	10‐12 min^[^ ^36^ ^]^	N/A	N/A	5 days	up to 0.5m in height^[^ ^18^ ^]^	annual, perennial^[^ ^18^ ^]^
Sensitive plant (*Mimosa pudica*)	pinnule	turgor	nastic movement	photonasty, nyctinasty	light, circadian clock	bending (open)	N/A	300‐3000 s^[^ ^37^ ^]^	N/A	N/A	5 days	leaflets 0.6–1.2 cm long, 0.3–0.4 cm broad^[^ ^18^ ^]^	annual, perennial^[^ ^18^ ^]^
Silk tree (*Albizia julibrissin*)	pinnule	turgor	nastic movement	photonasty, nyctinasty	light, circadian clock	bending (open)	100‐130°^[^ ^38^ ^]^	3 h^[^ ^38^ ^]^	33‐43°/h^*1^	N/A	N/A	5‐16 m in height^[^ ^23^ ^]^	perennial^[^ ^19^ ^]^
Sunflower (*Helianthus annuus*)	stem	growth	tropism	phototropism, heliotropismv	light, circadian clock	bending	10‐80° (12 h)^[^ ^39^, ^40^ ^]^	12 h^[^ ^39^, ^40^ ^]^	0.8‐6.7°/h^*1^	N/A	N/A	5‐20 ft in height, the head 3–24 in^[^ ^24^ ^]^	annual
Tomato *(Solanum lycopersicum L.)*	stem	turgor	nastic movement	photonasty	light	bending	220‐480° (1 day)	5 days	220‐480°/day^*1^	N/A	4 days	at least 4.5 mm in height	perennial
*Gentiana scabra*	flower	turgor	nastic movement	thermonasty, photonasty	temperature, light	bending (open)	15‐25° (60 min)^[^ ^41^ ^]^	60 min^[^ ^41^ ^]^	15‐25°/h^*1^	N/A	N/A	N/A	perennial
*Gentiana rhaetica*	flower	turgor	nastic movement	thermonasty, photonasty	temperature, light	bending (open)	N/A	3 min^[^ ^42^ ^]^	N/A	N/A	N/A	N/A	perennial
*Oxalis martiana*	flower	turgor	nastic movement	thermonasty, photonasty	temperature, light	bending (open)	N/A	3 h^[^ ^43^ ^]^	N/A	N/A	N/A	N/A	perennial
Dandelion (*Taraxacum officinale*)	flower	turgor	nastic movement	thermonasty, photonasty	temperature, light	bending (open)	50‐100°^[^ ^44^ ^]^	6 h^[^ ^44^ ^]^	8‐16°/h^*1^	N/A	9.1‐11.8 days^[^ ^29^ ^]^	5‐50 cm in height^[^ ^16^ ^]^	perennial^[^ ^16^ ^]^
Bushkiller (*Cayratia japonica*)	tendril	growth	tropism	thigmotropism	mechanical	coiling	15‐40° (12 min),^[^ ^25^ ^]^ 0–180° (30 min)^[^ ^26^ ^]^	more than 5h^[^ ^26^ ^]^	6°/min^*1^	N/A	N/A	at least 20 cm in height^[^ ^25^, ^26^ ^]^	perennial
Passion flower (*Passiflora caerulea*)	tendril	growth	tropism	thigmotropism	mechanical	coiling	N/A	more than 14.2 days^[^ ^66^ ^]^	N/A	6‐140 mN^[^ ^66^ ^]^	N/A	15–20 m^[^ ^45^ ^]^	perennial^[^ ^45^ ^]^
Venus flytrap (*Dionaea muscipula*)	leaf	turgor	nastic movement	thigmonasty	mechanical, electrical	bending (close)	50‐90°^[^ ^80^ ^]^	0.1‐0.8 s^[^ ^67^, ^74^ ^]^	10‐20 cm /s^[^ ^67^ ^]^	0.14 N^[^ ^67^ ^]^	N/A	up to 20 cm^[^ ^46^ ^]^	perennial^[^ ^46^ ^]^
Round‐leaved sundew (*Drosera rotundifolia*)	tentacle	turgor	nastic movement	thigmonasty	mechanical	bending (wrap)	N/A	10.0‐75.8 s^[^ ^70^ ^]^	0.3‐2.3° /s^[^ ^70^ ^]^	N/A	2‐3 months to flower, 150 days to germinate^[^ ^63^ ^]^	branches 2–44 mm^[^ ^47^ ^]^	perennial^[^ ^47^ ^]^
*Drosera tokaiensis*	flower	turgor	nastic movement	thigmonasty	mechanical	bending (close)	0‐45°^[^ ^48^ ^]^	2‐10 min^[^ ^48^, ^51^ ^]^	0‐22.5°/min^*1^	N/A	1‐2 months to flower, 100–150 days to germinate^[^ ^63^ ^]^	flower stalk 14.8 ± 5.59 cm^[^ ^51^ ^]^	perennial^[^ ^48^ ^]^
Sensitive plant (*Mimosa pudica*)	petiole	turgor	nastic movement	thigmonasty, seismonasty	mechanical, vibration, electrical	bending (fall)	25‐100°^[^ ^36^ ^]^	few sec^[^ ^36^ ^]^	N/A	15.82 ± 0.7 mN^[^ ^68^ ^]^	5 days	up to 0.5m in height^[^ ^18^ ^]^	annual, perennial^[^ ^18^ ^]^
Sensitive plant (*Mimosa pudica*)	pinnule	turgor	nastic movement	thigmonasty, seismonasty	mechanical, vibration, electrical	bending (close)	10‐80°^[^ ^75^, ^76^ ^]^	few sec^[^ ^75^, ^76^ ^]^	N/A	0.01‐0.4 mN^[^ ^37^ ^]^	5 days	leaflets 0.6–1.2 cm long, 0.3–0.4 cm broad^[^ ^18^ ^]^	annual, perennial^[^ ^18^ ^]^
Telegraph plant (*Codariocalyx motorius*)	leaf	turgor	nastic movement	thigmonasty, seismonasty	mechanical, vibration, sound	bending	N/A	N/A	N/A	N/A	3 months^[^ ^52^ ^]^	30.13‐41.00 cm in height^[^ ^52^ ^]^	perennial
Pine cone	scale	passive‐structual	passive	hygroscopic movement	humidity	bending	50‐75°^[^ ^77^, ^81^, ^82^ ^]^	30‐70 min (wet),^[^ ^69^, ^77^ ^]^ 350 min (dry)^[^ ^77^ ^]^	N/A	2.2‐3.6 N^[^ ^69^ ^]^	1 year for cone growth^[^ ^53^ ^]^	cone 8–15 cm^[^ ^53^ ^]^	perennial
Ice plant seed capsules (*Delosperma nakurense*)	seed capsule	passive‐structual	passive	hygroscopic movement	humidity	bending	N/A	few min^[^ ^78^ ^]^	N/A	N/A	18‐35 weeks^[^ ^54^ ^]^	seed capsules 1.5‐2.5 cm^[^ ^54^ ^]^	annual, perennial
Rose of Jericho (*Anastatica hierochuntica*)	skeleton	passive‐structual	passive	hygroscopic movement	water	bending	88% area increase^[^ ^49^ ^]^	2‐3 h^[^ ^49^ ^]^	N/A	N/A	3.5‐12 h to germinate^[^ ^64^ ^]^	skeletons 4–10 cm^[^ ^49^ ^]^	annual^[^ ^49^ ^]^
Horsetail spores (*Equisetum*)	elater	passive‐structual	passive	hygroscopic movement	humidity	extending	250‐350 µm^[^ ^55^, ^79^ ^]^	3.3‐3.6 s^[^ ^79^ ^]^	70‐106 µm/s^*1^	N/A	N/A	spores 50 µm^[^ ^55^ ^]^	perennial
Dandelion (*Taraxacum officinale*)	seed	passive‐structual	passive	pappus	potential energy	drifting	N/A	N/A	descent rate 26.9 ± 2.7 cm/ s^[^ ^56^ ^]^	N/A	seed dispersal starts 9–14 days after the start of flowering^[^ ^65^ ^]^	pappi 6.2‐7.4 mm,^[^ ^56^, ^57^ ^]^ stalk 80±10 mm^[^ ^56^ ^]^	perennial
Silver maple (*Acer saccharinum*)	seed	passive‐structual	passive	samara	potential energy	spinning	N/A	N/A	descent rate 0.55‐1.77 m/s^[^ ^58^ ^]^	N/A	N/A	fruit area 28–95 mm^2[^ ^58^ ^]^	perennial
Japanese maple (*Acer palmatum*)	seed	passive‐structual	passive	samara	potential energy	spinning	N/A	N/A	descent rate 1.2 m /s, spinning rate 130.9 rad/s^[^ ^71^ ^]^	N/A	N/A	wing 1.25‐2.90 cm^[^ ^59^ ^]^	perennial
Javan cucumber (*Alsomitra macrocarpa*)	seed	passive‐structual	passive	wing	potential energy	gliding	37 m^[^ ^72^ ^]^	24 s^[^ ^72^ ^]^	1.2‐2.1 m s^−1^, descent rate 0.3‐0.7 m/s^[^ ^72^ ^]^	N/A	N/A	wing 13–15 cm^[^ ^57^ ^]^	perennial
Sandbox tree (*Hura crepitans*)	seed	passive‐structual	passive	dry	humidity	explosion	0‐40 m^[^ ^60^ ^]^	N/A	13‐70 m /s^[^ ^60^, ^73^ ^]^	N/A	N/A	capsule 50–80 mm, seeds 15–25 mm, 11.2 m in height^[^ ^60^ ^]^	perennial
Squirting cucumber (*Ecballium elaterium*)	seed	turgor	passive	water pressure	water, mechanical	launch	5‐10 m^[^ ^61^ ^]^	30 ms^[^ ^61^ ^]^	20 m /s (max),^[^ ^61^ ^]^ 2.69‐3.06 m/s (terminal)^[^ ^62^ ^]^	N/A	N/A	fruit 3–4 cm,^[^ ^61^ ^]^ seeds 4.72‐5.02 mm^[^ ^62^ ^]^	perennial^[^ ^50^ ^]^

Notes. Passive‐Structural = motion generated by the release of elastic or hygromorphic energy stored in the plant's anatomical structure; no metabolic (growth or turgor) activity is involved.

*1 Values are estimated by the authors from the published data.

### 2.1 Growth

**Figure 2:**
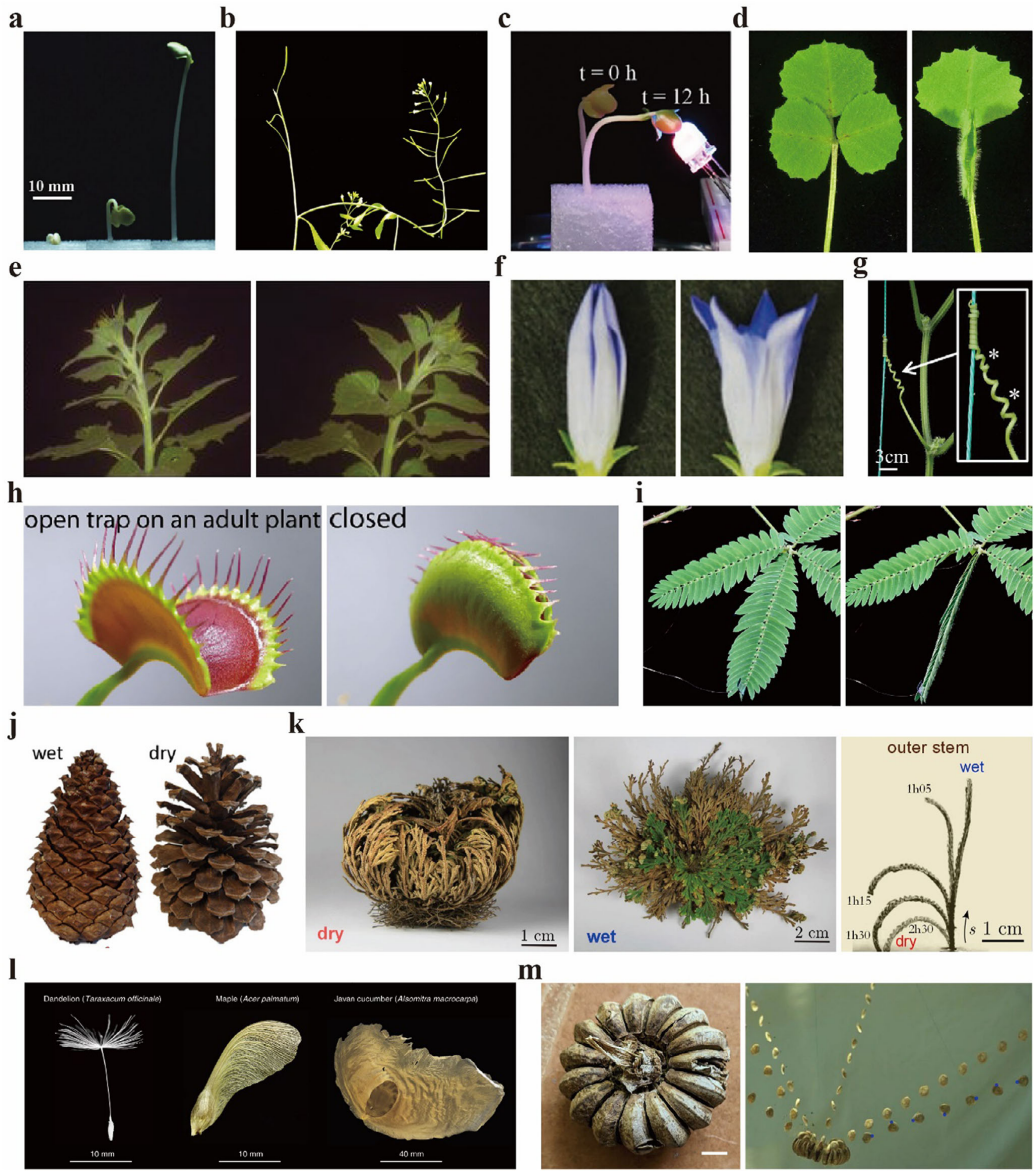
Examples of plant movements. a) Growth‐induced deformation (radish sprouts). Reproduced with permission.^[^ ^20^ ^]^ Copyright 2024, the authors. Published under CC‐BY license. b) Gravitropic bending of the stem (thale cress). Reproduced with permission.^[^ ^83^ ^]^ Copyright 2013, the authors. Published under CC‐BY license. c) Phototropic bending (radish sprouts). Reproduced with permission.^[^ ^20^ ^]^ Copyright 2013, the authors. Published under CC‐BY license. d) Diurnal leaf opening and closing driven by the circadian clock (*Medicago truncatula*). Reproduced with permission.^[^ ^84^ ^]^ Copyright 2023, John Wiley & Sons. e) Heliotropic movement of the floral head (sunflower). Reproduced with permission.^[^ ^39^ ^]^ Copyright 2016, American Association for the Advancement of Science. f) Flower opening and closing in response to diurnal temperature fluctuations (*Gentiana scabra*). Reproduced with permission.^[^ ^41^ ^]^ Copyright 2022, Oxford University Press. g) Twining movement of tendrils in response to mechanical stimulation (cucumber). Reproduced with permission.^[^ ^85^ ^]^ Copyright 2024, John Wiley & Sons. h) Leaf closure triggered by mechanical contact (Venus flytrap). Reproduced with permission.^[^ ^86^ ^]^ Copyright 2022, the authors. Published under CC‐BY license. i) Leaf closure triggered by electrical stimulation (sensitive plant). j) Hygroscopic movement of scales – they close in humid conditions and open in dry conditions (pine cone). Reproduced with permission.^[^ ^87^ ^]^ Copyright 2021, the authors. Published under CC‐BY license. k) Hygroscopic movement of leaves – they close in dry conditions and open in humid conditions (The Rose of Jericho). Reproduced with permission.^[^ ^88^ ^]^ Copyright 2022, the authors. Published under CC‐BY license. l) Seeds adapted for airborne dispersal (from left: dandelion, Japanese maple, Javan cucumber). Reproduced with permission.^[^ ^89^ ^]^ Copyright 2022, Cell Press. m) Seed dispersal via capsule explosion (sandbox tree). Reproduced with permission.^[^ ^73^ ^]^ Copyright 2020, Oxford University Press.

Growth is one of the most important types of plant movement. Although growth may seem slow compared to the rapid movements of certain specialized organs—such as the leaf closure of the Venus flytrap—it can generate significant force over time. For instance, radish sprouts, as shown in **Figure** **2**a, have been reported to exhibit a displacement of 16.3–46.4 mm over a 40 h actuation period, achieving speeds of 0.4–1.2 mm h^−1^, and generating a force of 31.8–35.4 mN after germination.^[^ ^20^ ^]^ In contrast, dandelion growth can produce forces between 2–3 N,^[^ ^30^ ^]^ demonstrating that plants are capable of generating a wide range of mechanical forces—from millinewtons to newtons—depending on the species and environmental conditions. Among slow‐growing plants, Moso bamboo is notable for its vigorous growth, reaching heights of 9.67–17.5 m and growth rates of up to 114.5 cm per day,^[^ ^21^, ^31^ ^]^ exemplifying the power of plant‐driven motion over time.

### 2.2 Stimuli Response

#### 2.2.1 Gravity

In terrestrial habitats, virtually every land plant maintains its body orientation aligned with Earth's gravity vector, with shoots growing upward and roots penetrating downward. This phenomenon is due to gravitropism, the ability of plants to sense and respond to gravity.^[^ ^90^, ^91^, ^92^ ^]^ Through gravitropism, plants generate a restorative force that enables them to maintain or reestablish vertical orientation in alignment with the gravitational pull, as illustrated in Figure 2b. For example, when pea sprouts are placed horizontally, their stems bend and grow vertically, producing a bending force of ≈100 mN.^[^ ^93^ ^]^

#### 2.2.2 Light and Circadian Clock

Plants adjust their posture or position in order to capture more light.^[^ ^94^, ^95^ ^]^ As presented in Figure 2c, they sometimes bend their stems up to ≈90° in response to phototropism.^[^ ^20^ ^]^ Additionally, some plants exhibit photonasty, a type of movement in response to light independent of its direction. For example, species commonly found in the legume family—such as common bean, black locust, sensitive plant, and silk tree—change the angles of their leaves or branches between day and night, as depicted in Figure 2d.^[^ ^84^ ^]^ Reported angle changes include 89° over 7 h for common bean, 102° over 9 h for black locust, and 100–103° over 3 h for silk tree.^[^ ^34^, ^35^, ^38^ ^]^ The corresponding speeds are estimated at 13, 11, and 33–43 ° h^−1^, respectively. The sensitive plant requires 10–12 min for the petiole to rise and 5–50 min for the leaves to open.^[^ ^36^, ^37^ ^]^ These plants also possess a circadian clock, which enables them to maintain and repeat periodic movements associated with diurnal photonasty, a phenomenon known as nyctinasty. As a result, they can sustain these periodic movements for several days, even in constant darkness. Furthermore, sunflowers exhibit a well‐known example of heliotropism, in which their flower heads track the sun from east to west during the day and reorient toward the east at night, as displayed in Figure 2e.^[^ ^39^ ^]^ The bending angle is 10–80° over 12 h, with an estimated speed of 0.8–6.7° h^−1^.^[^ ^39^, ^40^ ^]^ In addition, the apical hook observed in dicotyledonous plants develops at the tip of the axis during the dark period. This structure protects the shoot apex from mechanical damage as the seed germinates and the seedling grows through the soil. When the seedling emerges above the soil, the hook opens in response to external light, allowing the shoot to straighten. However, in tomatoes, light exposure exaggerates the curvature, resulting in a curling response.^[^ ^96^, ^97^ ^]^ In these reports, bending angles of 220–480° per day have been observed, with actuation continuing for up to 5 days.

#### 2.2.3 Temperature

Plants are also highly sensitive to temperature changes and can modify the shape of their roots and flowers in response.^[^ ^42^, ^98^ ^]^ For example, corn exhibits thermotropism; its roots grow and curve in response to variations in soil temperature.^[^ ^99^ ^]^ Additionally, the flowers of *Gentiana scabra*, *Gentiana rhaetica*, *Oxalis martiana*, and dandelion exhibit thermonasty, opening and closing in response to daily temperature fluctuations, as indicated in Figure 2f.^[^ ^41^, ^42^, ^43^, ^44^ ^]^ This response is driven by growth movements, even though it falls under the category of nastic movements. *Gentiana scabra* has been reported to exhibit petal bending angle changes of 15–25° within 60 min, with an estimated speed of 15–25° h^−1^ when placed in an environment at 16–22 °C.^[^ ^41^ ^]^ The reported actuation time for *Gentiana rhaetica* is 3 min, while *Oxalis martiana* requires 3 h. Dandelion has a recorded actuation time exceeding 6 h, achieving a 50–100° change within 30 min, with an estimated speed of 8–16° h^−1^.^[^ ^44^ ^]^

#### 2.2.4 Physical Interaction

In addition, plants exhibit various responses to physical stimuli, such as touch, vibration, and sound. For instance, climbing plants such as grape, cucumber, bushkiller, and passion flower exhibit thigmotropism (the ability to coil around supports or neighboring plants in response to physical contact), as shown in Figure 2g.^[^ ^100^, ^101^, ^102^, ^103^ ^]^ Notably, bushkiller can coil from 0 to 180° within 30 min, corresponding to an estimated rate of 6° min^−1^.^[^ ^26^ ^]^ Moreover, passion flowers have been reported to generate coiling forces of 6–140 mN.^[^ ^66^ ^]^ Furthermore, plants such as Venus flytrap (Figure 2h), round‐leaved sundew, *Drosera tokaiensis*, and sensitive plant exhibit rapid movements—such as branch bending or leaf and flower closure—in response to mechanical stimuli, a phenomenon known as thigmonasty.^[^ ^48^, ^67^, ^75^, ^103^, ^104^ ^]^ The sensitive plant has been reported to achieve leaf closures of 10–80° and generate forces of 0.01–0.4 mN within seconds.^[^ ^37^, ^75^, ^76^ ^]^ Whereas the Venus flytrap can achieve leaf closures of 50–90° and generate a significantly stronger force of ≈0.14 N with 0.1‐0.8 s.^[^ ^67^, ^74^, ^80^ ^]^ *Drosera tokaiensis* bends its flowers by 0–45° over 2–10 min, with an estimated speed of 0–22.5° min^−1^.^[^ ^48^, ^51^ ^]^ Additionally, some thigmonastic plants can detect and respond to vibrations, a response known as seismonasty, with the sensitive plant being a well‐known example. Moreover, it has been confirmed that the telegraph plant responds to mechanical contact, vibration, and even sound by moving its leaves.^[^ ^52^ ^]^

#### 2.2.5 Electrical Signals

Some plants also respond to electrical stimuli. For example, the roots of plants such as thale cress and maize exhibit electrotropism, altering their growth direction in response to electric fields.^[^ ^105^, ^106^ ^]^ Additionally, both the Venus flytrap and sensitive plant (Figure 2i) close their leaves in response to both physical and electrical stimuli.^[^ ^36^, ^37^, ^107^, ^108^, ^109^, ^110^ ^]^ The characteristics of these plants were previously described in Section 2.2.4. Plants that respond to electrical input offer unique advantages for seamless integration with existing electronic systems. They can function as alternatives to traditional electronic components, reducing or even eliminating the need for plant‐specific interface technologies.

#### 2.2.6 Humidity and Water

Some plant species undergo reversible structural changes in response to variations in ambient humidity or internal water content, enabling them to disperse seeds or spores and protect their structures. For example, pine cones open their scales under dry conditions, facilitating seed release (Figure 2j).^[^ ^69^, ^81^, ^82^ ^]^ Conversely, as humidity rises, the scales close again to protect the inner tissues. It takes 30–70 min to absorb the moisture and 350 min to dry. The reported bending angle ranges from 50–75°, with generated forces between 2.2‐3.6 N.^[^ ^69^, ^77^, ^81^, ^82^ ^]^ In contrast, ice plant seed capsules close their outer layers when dry and open them as humidity increases, demonstrating species‐dependent response patterns.^[^ ^78^ ^]^ These movements, triggered by the swelling and shrinking of cell walls due to moisture, are known as hygroscopic movements. The Rose of Jericho curls its leaves into a spherical shape during dry periods, facilitating easy transport.^[^ ^49^ ^]^ Upon absorbing water, it re‐expands, and its leaves unfurl (Figure 2k).^[^ ^88^ ^]^ This response indicates an 88% increase in surface area occurring over 2–3 h. On a microscale, horsetail spores exhibit walking‐ or jumping‐like movements through repeated cycles of drying and rehydration, underscoring the remarkable diversity of plant responses to humidity changes.^[^ ^55^, ^79^ ^]^ Reported measurements show an elongation of 250–350 µm over 3.3‐3.6 s, with an estimated speed of 70–106 µm s^−1^.

### 2.3 Seed Dispersal

Seed dispersal also takes on a wide range of forms (Figure 2l).^[^ ^89^ ^]^ For example, dandelions use pappi—feathery structures at the top of their seeds—to catch the wind, allowing them to remain airborne for extended periods and travel long distances. Their descent speed has been reported as 26.9 m s^−1^.^[^ ^56^ ^]^ Meanwhile, silver maple and Japanese maple produce winged fruits called samaras, which spin as they fall, enabling slower descent and broader seed dispersal.^[^ ^58^, ^59^, ^71^ ^]^ For silver maple, descent speeds range between 0.55–1.77 m s^−1^,^[^ ^58^ ^]^ and for Japanese maple, the descent rate is 1.2 m s^−1^.^[^ ^71^ ^]^ Moreover, ​the Javan cucumber features a large, thin wing and demonstrates gliding behavior from elevated locations, with recorded flight distances of up to 37 m over 24 s, at speeds of 1.2–2.1 m s^−1^.^[^ ^72^ ^]^ On the other hand, some plants eject seeds by rapidly releasing the pressure that builds up within the fruit. The Sandbox tree's seed capsules burst as they dry (Figure 2m), a process that also exemplifies hygroscopic movement.^[^ ^73^ ^]^ The seeds can be dispersed over distances of 0–40 m at speeds of 13–70 m s^−1^.^[^ ^60^, ^73^ ^]^ The squirting cucumber stores water internally, ejecting its seeds when triggered by mechanical stimulation.^[^ ^61^ ^]^ The seeds are dispersed 5–10 m at speeds of 20 m s^−1^.^[^ ^61^ ^]^

## 3 Technologies for Controlling Plant Movements

**Figure 3:**
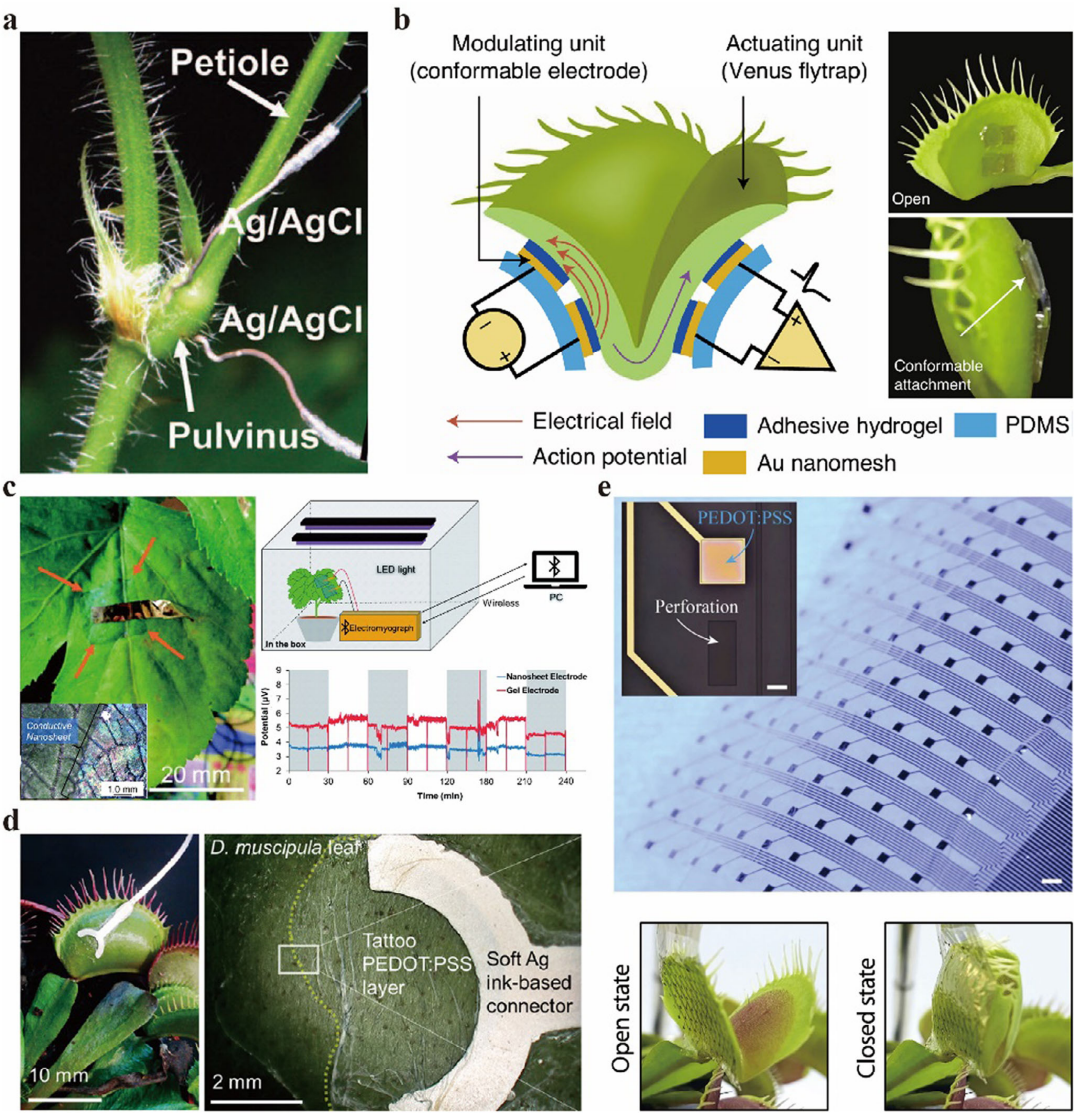
Electrodes designed for plant attachment. a) Invasively connected electrodes for driving *Mimosa pudica* pulvinus. Reproduced with permission.^[^ ^36^ ^]^ Copyright 2010, Taylor & Francis. b) Electrodes attached to opposite lobes of a Venus Flytrap for stimulation and action potential recording. Reproduced with permission.^[^ ^107^ ^]^ Copyright 2021, Springer Nature. c) The conductive nanosheets and the biopotential changes of a plant leaf during the turning on and off of the LED light. Reproduced with permission.^[^ ^113^ ^]^ Copyright 2024, the authors. Published under CC‐BY license. d) The inkjet‐printed electrode. Reproduced with permission.^[^ ^114^ ^]^ Copyright 2021, the authors. Published under CC‐BY license. e) The multielectrode array. Reproduced with permission.^[^ ^115^ ^]^ Copyright 2023, the authors. Published under CC‐BY license.

Given that most of the stimuli discussed in Section 2, such as light, temperature, physical interaction, electricity, humidity, and water, can be regulated using electrical components, electrical technologies are likely to become a mainstream method for controlling plant movements. For instance, a liquid alloy electrode printed onto a bean sprout successfully altered its growth direction by powering an overhead LED.^[^ ^111^ ^]^ For this reason, this section explores available electrical technologies compatible with plant systems. Among these, invasive methods, such as inserting wires and needles, are the most direct approach (**Figure** **3**a).^[^ ^36^ ^]^ However, these invariably damage plant tissues, potentially reducing both plant lifespan and movement repeatability. Additionally, depending on placement, inserted electrodes may interfere with natural plant movement.

Alternatively, thin, film‐like flexible electrodes that can be directly attached to plant surfaces offer a non‐invasive solution that preserves movement.^[^ ^112^ ^]^ An example is depicted in Figure 3b, where thin‐film electrodes made of hydrogel layers, gold (Au), and polydimethylsiloxane (PDMS) are attached to Venus flytrap leaves to generate an electric potential that triggers leaf closure.^[^ ^107^ ^]^ Various materials and structural designs are also available for plant‐compatible, attachable electrodes. A conductive nanosheet made of poly(3,4‐ethylenedioxythiophene): polystyrene sulfonate (PEDOT: PSS) coated onto a poly(styrene‐b‐butadiene‐b‐styrene) (SBS) nanosheet successfully detected biopotential changes in *Angelica keiskei* leaves. This nanosheet adhered to uneven surfaces like leaf veins without chemical adhesives, thanks to its ultrathin thickness (300 nm) and lightweight (150 µg) (Figure 3c).^[^ ^113^ ^]^ Tattoo electrodes fabricated via inkjet printing of PEDOT: PSS onto a 2‐µm‐thick tattoo substrate measured the biopotential of Venus flytraps (*D. muscipula*) during mechanical stimulation of their trigger hairs over a 10‐day period (Figure 3d).^[^ ^114^ ^]^ A multielectrode array produced using photolithography techniques enabled large‐scale, high‐resolution mapping of biopotentials in Venus flytrap traps (Figure 3e).^[^ ^115^ ^]^

While the above discussion focused on plant specific‐applications, many other types of thin, film‐like electrodes exist, and readers are encouraged to consult the literature for further information.^[^ ^116^, ^117^, ^118^ ^]^

## 4 Plant‐Based Robotic Systems

As described in the previous section, plants possess inherent mechanical responses that can be harnessed to function as actuators. In fact, numerous studies have integrated plants into robotic systems to enable functions essential for tasks such as locomotion, object gripping, and switching. This section categorizes existing studies into two types and highlights their key characteristics: those powered by plant growth and those activated by external stimuli.

**Figure 4:**
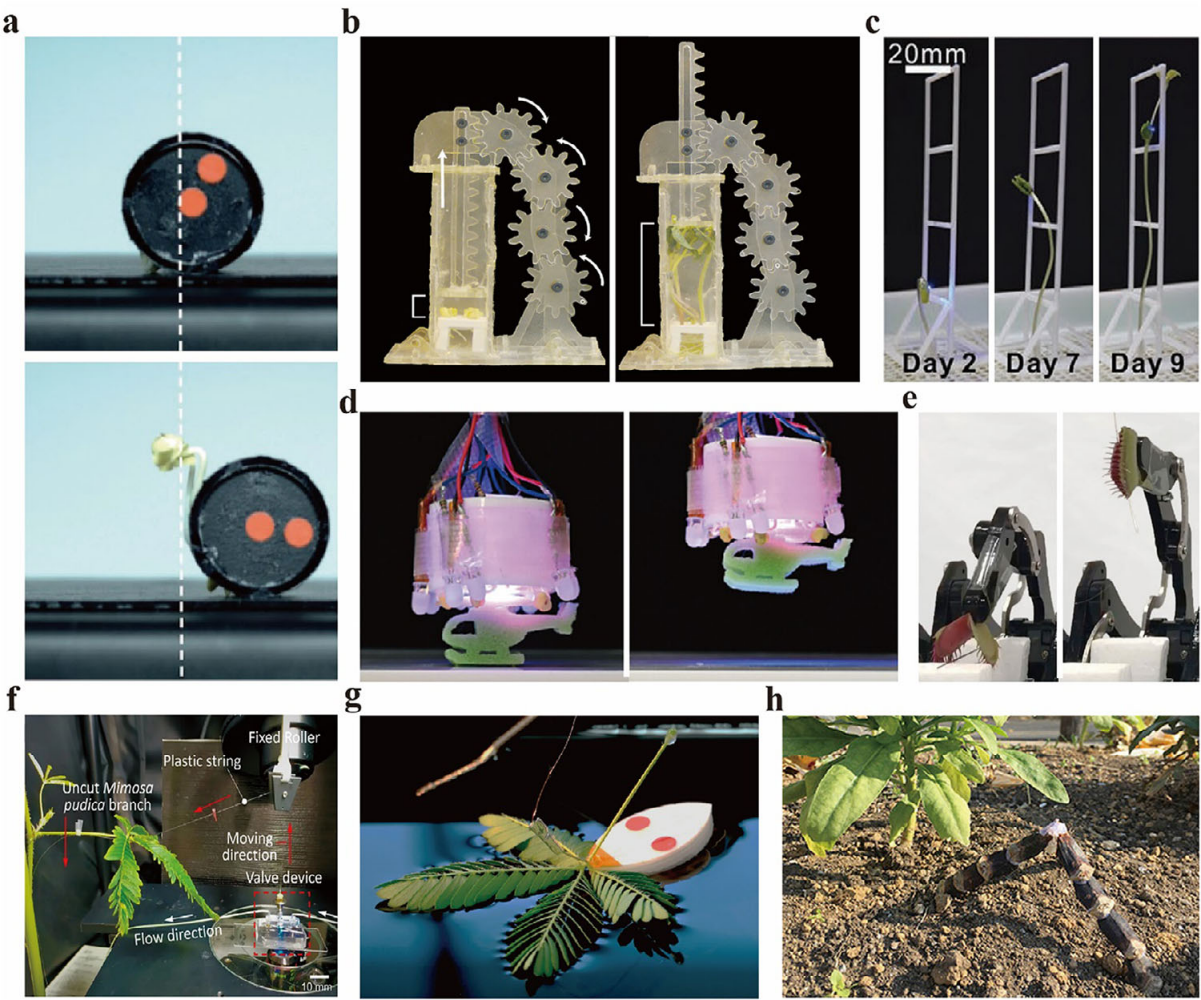
Examples of plant‐powered robots, devices, and systems. a) The mobile robot converts the growth‐induced displacement into rotational motion. Reproduced with permission.^[^ ^20^ ^]^ Copyright 2024, the authors. Published under CC‐BY license. b) The gear that rotates due to growth‐induced deformation. Reproduced with permission.^[^ ^119^ ^]^ Copyright 2024, the authors. Published under CC‐BY license. c) Controlling the growth trajectory using an LED attached to the tip of a plant. Reproduced with permission.^[^ ^111^ ^]^ Copyright 2020, John Wiley & Sons. d) Light‐guided plants grasp an object. Reproduced with permission.^[^ ^20^ ^]^ Copyright 2024, the authors. Published under CC‐BY license. e) The Venus flytrap gripper that operates in response to electrical stimulation. Reproduced with permission.^[^ ^107^ ^]^ Copyright 2021, Springer Nature. f) Microfluidic control using a sensitive plant activated through mechanical stimulation. Reproduced with permission.^[^ ^68^ ^]^ Copyright 2022, the authors. Published under CC‐BY license. g) The mobile robot operating on the water surface, utilizing the electrical response of a sensitive plant. h) A height‐changing robot that utilizes the hygroscopic deformation of a pine cone. Reproduced with permission.^[^ ^77^ ^]^ Copyright 2020 by the Massachusetts Institute of Technology. All rights reserved.

In the first category, robots convert plant growth into mechanical output. In the robot displayed in **Figure** **4**a, the linear elongation of radish sprouts sequentially pushes the ground, resulting in a rotation motion and achieving a displacement of 14.6 mm at an average speed of 0.8 mm h^−1^.^[^ ^20^ ^]^ In the device indicated in Figure 4b, the growth force of plants is converted into rotational motion by driving a set of pinion gears via a rack, successfully demonstrating the integration of plants into a conventional mechanical transmission system.^[^ ^119^ ^]^ In addition, the same study presents several robotic devices that leverage plant growth to achieve functions such as texture and morphological changes. A key advantage of devices powered by plant growth is their ability to function entirely through the plant's intrinsic biological processes, without the need for external power sources such as electricity.

In the second category, robots utilize plant responses to external stimuli. In Figure 4c, LEDs attached to the plant tip to act as a light source, inducing phototropism and controlling the plant's growth direction.^[^ ^111^ ^]^ A phototropism‐based gripper made from radish sprouts (Figure 4d) is capable of gripping and releasing a 0.1 g object.^[^ ^20^ ^]^ The Venus flytrap gripper presented in Figure 4e, actuated by electrical stimulation, can firmly grasp a 1 g object.^[^ ^107^ ^]^ Additionally, a microfluidic valve that utilizes the mechanical response of the sensitive plant's petiole has been developed (Figure 4f).^[^ ^68^ ^]^ Using the same plant, a robot capable of locomotion on the water surface, driven by the electrical stimuli, achieved a maximum velocity of 3.3 × 10^−5^ m s^−1^ and a thrust of 52.3 mN (Figure 4g).^[^ ^37^ ^]^ Lastly, the device shown in Figure 4h uses the moisture‐induced deformation of a pine cone to form a height‐adjustable device, achieving a displacement of 49 mm.^[^ ^77^ ^]^ These stimuli‐responsive devices hold promise for integration with conventional electronic systems through the artificial modulation of applied stimuli.

These examples of robotic systems collectively highlight the potential of leveraging plant actuation characteristics as alternatives to traditional actuators. However, they remain largely at the proof‐of‐concept, representing early development efforts and leaving significant room for future research aimed at realizing practical applications.

## 5 Outlook

As revealed by the survey presented in this article summarized in Table 1, it is evident that current knowledge of plant actuation characteristics, such as displacement and generated force, remains fragmented and limited. In addition to other actuation characteristics, such as velocity and repeatability, fundamental material properties, including elasticity and density, are essential for the conceptualization and design of plant‐based robots. However, these data have not been systematically gathered or documented. Therefore, developing a comprehensive database of plant actuation characteristics and material properties should be a high priority for future research. Such a database, combined with standardized experimental conditions and protocols, e.g., consistent temperature, humidity, and light intensity for different species, would greatly enhance the reproducibility and reliability of experiments. Establishing this knowledge base, along with clear evaluation guidelines, would provide a solid foundation for treating plants as robotic actuators, thereby accelerating advances in plant robotics.

Furthermore, the performance values presented in Table 1 appear modest. One strategy to address this limitation is to employ multiple plants in parallel to achieve enhanced performance. In a previous work, object grasping using a gripper composed of multiple radish sprouts was demonstrated^[^ ^20^ ^]^ This highlights that the flexibility of individual plants allows them to act cooperatively, performing advanced tasks that would be impossible for a single plant. Another factor contributing to the low performance values in Table 1 is the predominance of herbaceous species in existing studies. In contrast, woody plants, such as trees, undergo lignification over time, resulting in superior material properties compared to herbaceous plants. This suggests that larger, lignified plants represent a promising resource for actuating materials, enabling the potential for scaling up plant‐based robots.

Analytical and simulation models play a crucial role in the design of robots that harness the diverse characteristics of plant movements. Although rapid prototyping with real plants can offer valuable design insights, plant germination and growth often require extended periods of time, making it difficult to maintain realistic experimental timelines. This challenge is especially pronounced when working with lignifying plant species, which may require several years of growth. This situation also motivates alternative approaches, such as replacing real plants with artificial plant‐mimetic elements like soft actuators.^[^ ^120^, ^121^, ^122^ ^]^ However, replicating key characteristics of living plants, such as self‐growing, remains technically challenging and is subject to numerous constraints. Meanwhile, research on modeling techniques and computational tools for simulating plant growth and stimuli‐responsive movements has been advancing.^[^ ^80^, ^123^, ^124^, ^125^, ^126^, ^127^, ^128^ ^]^ With support from the techniques available in the literature, a robust modeling and simulation framework can streamline the design process and identify optimal solutions before committing to time‐consuming and uncertain biological experiments. Ultimately, combining simulations with targeted experiments can greatly accelerate robotic design while capturing the unique dynamic behaviors of living plant actuators.

The majority of current research on controlling plant movement has concentrated on external factors, such as light or electrical stimulation. However, approaches that utilize signaling molecules, such as plant hormones and water‐based osmotic regulation, are also promising and warrant further exploration.^[^ ^129^, ^130^, ^131^ ^]^ Plant hormones include various compounds, such as auxins, cytokinins, and gibberellins, each exerting specific physiological effects, including the regulation of cell elongation, division, and maturation.^[^ ^132^ ^]^ By locally manipulating these chemicals at the cellular level, it may be possible to induce diverse morphological changes in targeted regions, such as swelling, bending, and twisting. Furthermore, biostimulants, gaining increasing attention in recent years^[^ ^133^, ^134^, ^135^ ^]^ contain amino acids, organic acids, and microbial metabolites, and are reported to enhance plant growth and metabolism through multiple pathways. These substances may also aid in controlling morphogenesis, in addition to supporting growth and stress responses, opening new avenues for their application in plant robotics. Looking ahead, it will be important to systematically investigate the mechanisms of action, efficacy, and persistence of different chemical agents while integrating them with technologies such as microfluidic devices and injection systems in order to achieve more precise and selective chemical control. In addition to these chemical strategies, another promising approach is to enhance the robot's core capabilities through genome editing. This technique could enable the identification and regulation of genes encoding proteins associated with movement, or could strengthen the plant's barrier functions against external threats. Such genetic approaches could allow precise control over plant responses, something that is difficult to achieve through electrical or mechanical stimulation alone. Moreover, they have the potential to enhance not only deformation but also characteristics such as speed and repeatability, thereby broadening the scope of applications in plant robotics. As this line of research is also expected to deepen the understanding of plant physiology, it encourages collaboration between plant biologists and engineering researchers.

Because plant‐based robots are intended to promote sustainability, their structural materials (the robotic body), should ideally be biodegradable. Accordingly, introducing biodegradability into electrodes and other related components remains an important challenge. In this context, a wide range of biodegradable materials has been reported in the literature.^[^ ^8^, ^136^, ^137^, ^138^ ^]^ Moreover, in the fields of sustainable robotics and electronics, numerous biodegradable components, such as sensors, processors, and batteries, have been developed.^[^ ^8^, ^136^, ^137^, ^138^ ^]^ Leveraging these technologies represents one of the most promising strategies for realizing fully biodegradable, autonomous plant robots capable of operating in a closed‐loop fashion. In parallel, efforts to replace conventional robotic components with living plant parts are gradually progressing,^[^ ^14^ ^]^ and in the future, it may become feasible to create robotic systems composed entirely of plant materials. Furthermore, selecting and breeding plant species optimized for use in plant robots is considered a promising direction for future research. In addition to conventional breeding improvement goals, such as disease resistance, stress tolerance, and harvest suitability, future efforts that emphasize actuation traits, including strong germination forces, specific motion responsiveness, and adaptable morphological control, are expected to further enhance the performance of plant‐based robotic systems.

Field testing is essential in the development of plant‐based robots to validate their performance and functionality in natural environments. It also helps identify challenges and unique characteristics of robots powered by living plants in long‐term operation. In particular, because plant growth and movement are highly dependent on environmental factors, such as temperature, light, humidity, and wind, it is crucial to systematically understand the influence of these variables. Moreover, the potential impact of pests and microorganisms must be considered, as these biological factors can affect plant performance plants, and, by extension, robot functionality. An additional point that warrants attention is that, as mentioned earlier, testing may span several years depending on the plant species, making experimental validation difficult to align with standard development timelines. Although simulation environments are useful in the design stage, they may inherently struggle to reproduce real‐world weather conditions and sudden environmental fluctuations such as heat waves, strong winds, or heavy rainfall. Therefore, moving forward, it is important to integrate findings from both simulations and field tests. Conducting trials across diverse field settings, including agricultural fields, urban greening areas, and natural environments, will help establish environmentally robust plant robots, along with their operational strategies and performance metrics.

The research efforts discussed thus far enable envisioning of a wide range of plant robotic applications, as illustrated in Figure 1. One vision is to leverage the self‐growing capabilities of plants to gradually form or reinforce structures such as bridges, walls, or erosion barriers, thereby contributing to sustainable infrastructure and disaster prevention, for example, living retaining walls for landslides mitigation. Another potential application involves object transportation or actions such as harvesting and capturing, driven by plant motion and growth, which could serve as novel autonomous mechanisms in agriculture or environmental management. The inherent sensitivity of plants to environmental changes makes them promising candidates for biosensors; plant‐based mobile robots could monitor soil conditions, water quality, or pollutants by signaling as physiological response signals. In ecological restoration, plant robots could assist with reforestation or greening efforts by actively planting themselves or dispersing seeds, thereby integrating robots with natural regeneration processes.

The most intriguing aspect of these plant robotic systems is their paradigm‐shifting mode of operation; they function on extremely slow timescales, often so slow that they appear static to the human eye. This contrasts sharply with traditional robotics, which typically prioritizes speed and rapid deployment. This quiet, slow mode of operation may represent the very essence of plant robotics. Such systems can perform tasks sustainably and persistently, blending seamlessly into their environment without drawing attention from humans or other organisms. Their extremely slow dynamics enable integration into local ecosystems, minimizing environmental disruption. In a sense, they are “more alive”, functioning as living grasses or forests that can accomplish tasks that extend beyond conventional expectations. In this way, plant robotics has the potential to redefine how robots themselves are perceived, opening new pathways for sustainable and environmentally friendly robotics.

## Conflict of Interest

The authors declare no conflict of interest.

## Data Availability

The data supporting the findings of this study is available from the corresponding author upon reasonable request.
